# Intravesicular epidermal growth factor receptor subject to retrograde trafficking drives epidermal growth factor-dependent migration

**DOI:** 10.18632/oncotarget.23766

**Published:** 2017-12-29

**Authors:** Sabrina Maisel, Derrick Broka, Joyce Schroeder

**Affiliations:** ^1^ Cancer Biology Graduate Interdisciplinary Program, University of Arizona, Tucson, AZ, USA; ^2^ Arizona Cancer Center, University of Arizona, Tucson, AZ, USA; ^3^ Department of Molecular and Cellular Biology, University of Arizona, Tucson, AZ, USA; ^4^ BIO5 Institute, University of Arizona, Tucson, AZ, USA

**Keywords:** epidermal growth factor receptor, MUC1, cetuximab, migration, retrograde trafficking

## Abstract

The Epidermal Growth Factor Receptor (EGFR) is frequently mutated and overexpressed in metastatic cancer. Although EGFR is a transmembrane tyrosine kinase localized to the basolateral membrane in normal epithelium, it is frequently found intracellularly localized in transformed cells. We have previously demonstrated the epithelial adaptor protein mucin 1 (MUC1) alters trafficking of EGFR, inhibiting its degradation and promoting its translocation to the nucleus, where it can directly modulate gene transcription. Here, we demonstrate that MUC1 promotes the retention of EGF-bound EGFR in Early Endosome Antigen1 (EEA1)-positive vesicles while preventing its trafficking to the lysosome. These events result in the accumulation of endosomal vesicles harboring active receptor throughout the cell and a reorganization of the actin cytoskeleton. EGF-dependent cell migration and filopodia formation is reliant upon this altered trafficking, and can be prevented by blocking retrograde trafficking. Together, these results indicate that intracellular EGFR may play an essential role in cancer metastasis and a potential mechanism for the failure of therapeutic antibodies in EGFR-driven metastatic breast cancer.

## INTRODUCTION

Epidermal Growth Factor Receptor (EGFR), a receptor tyrosine kinase and member of the ERBB family, is overexpressed in cancers including glioblastoma, head and neck, bladder, non-small cell lung, and breast [1, 2]. When activated, EGFR regulates a variety of cellular processes, including survival, growth, migration, and adhesion [3]. EGFR expression is common in heterogeneous triple negative breast cancer (a cancer characterized by the lack of estrogen and progesterone hormone receptors and the HER2 receptor), and expression is associated with aggressive disease progression and poor survival rates [4, 5]. In normal tissue, ligand binding to a monomeric receptor on the basolateral membrane results in EGFR homo- and/or hetero-dimerization and transphosphorylation, providing active docking sites for numerous adaptor proteins. Once activated, EGFR primarily undergoes clathrin-mediated endocytosis where it maintains interactions with proteins such as Grb2 post-internalization, allowing for prolonged signal transduction from within endosomes [6, 7]. Other signaling pathways, including P-I3K and PLCγ, display increased activity once EGFR has become internalized [8], highlighting the location-dependent activation required to regulate EGFR signaling. Post-internalization, basolateral EGFR is predominantly trafficked through the early endosome to the late endosome, with the process terminating in the lysosome, resulting in EGFR degradation [6]. EGFR can also meet other fates, including recycling to the cell surface and retrotranslocation to the mitochondria and nucleus [9–11]. Retrotranslocation occurs when endocytosed transmembrane proteins such as EGFR become involved in the secretory or biosynthetic pathway, such as the trans-golgi network, the endoplasmic reticulum, and other pathways resulting in trafficking to and from the plasma membrane [12, 13]. Nuclear EGFR has been shown to traffic via retrotranslocation pathways to be delivered to the nucleus from the cell surface, similar to other proteins which have been shown to modulate downstream signal transduction from within endosomes while trafficking internally [14, 15]. Importantly, intracellular and nuclear EGFR is strongly correlated with poor therapeutic responses and metastatic progression in breast cancer, resulting in a 3.4 times greater mortality risk [16, 17].

MUC1 is a tumor antigen that can modulate EGFR activity, with EGF-dependent degradation of EGFR inhibited by co-expression of MUC1 [18]. We have previously demonstrated via the WAP-TGFα transgenic mouse model that EGFR is colocalized with the oncogenic adaptor protein MUC1 in hyperplastic and tumor tissues and EGFR-driven breast cancer is largely MUC1-dependent [19]. Furthermore, the ability of EGFR to drive Cyclin D1 expression in tumors as well as lung metastasis are both MUC1-dependent. Additional studies demonstrated that through interactions with MUC1, EGFR is not degraded upon ligand binding and instead is recycled and/or trafficked to the nucleus, where it interacts with chromatin directly, including the promoter of Cyclin D1 [19, 20]. Finally, MUC1 can also promote EGFR-dependent cell motility and acinar branching of breast cancer cells through the upregulation of c-Met [21].

In the present study, we investigated the mechanism by which MUC1 alters EGFR trafficking and drives metastatic progression. Here, we demonstrate that MUC1 inhibits the targeting of EGFR to the lysosome while sequestering EGFR in EEA1-positive vesicles. EGFR in this intracellular location remains active, leading to retrograde trafficking-dependent formation of filopodia and migration while limiting the efficacy of the anti-EGFR antibody Cetuximab.

## RESULTS

### MUC1 regulates trafficking of EGFR through EEA1-positive endosomes

**Figure 1:**
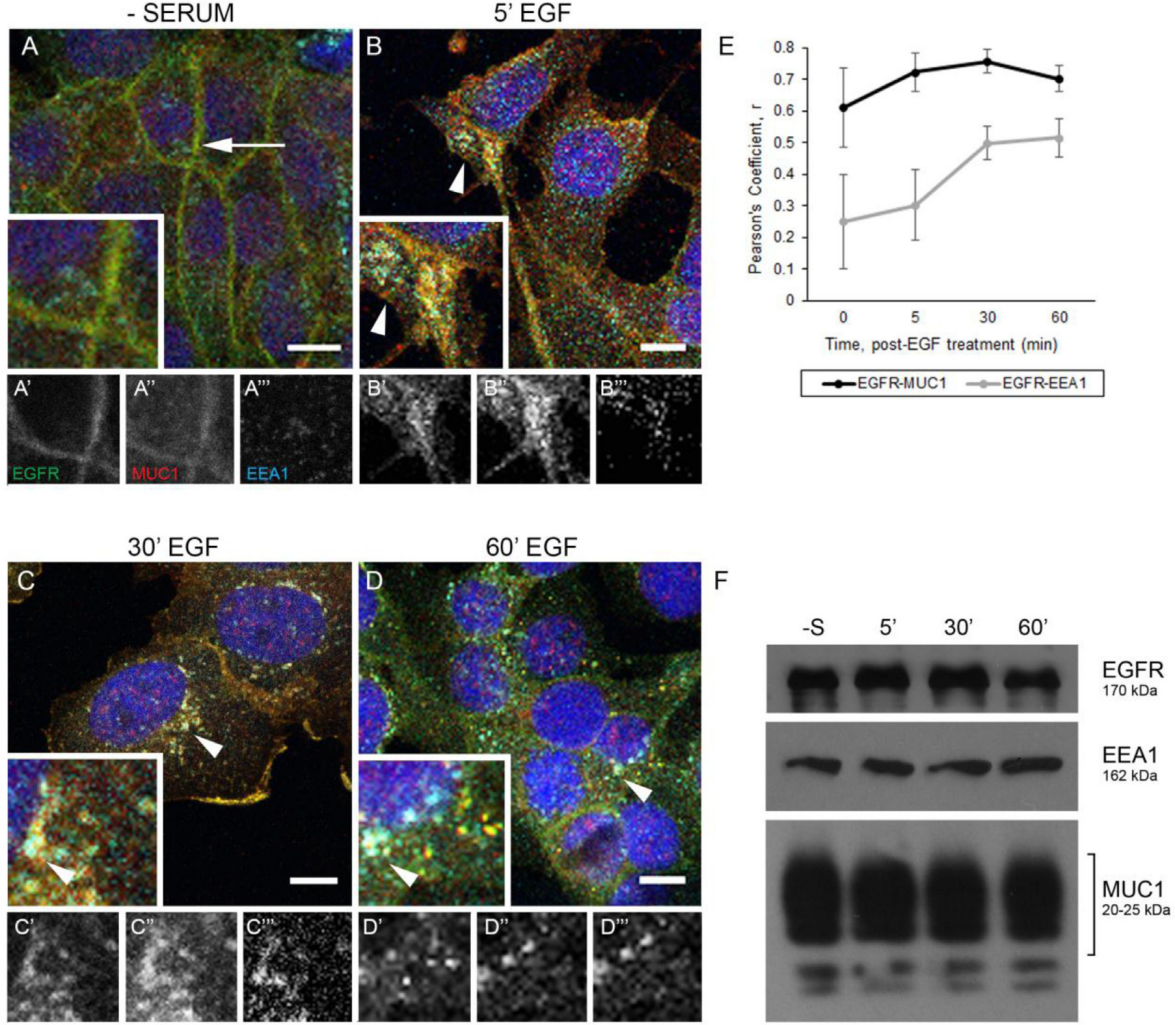
EGFR and MUC1 colocalize with early endosome markers (**A–D**) BT20cells were serum-starved overnight, treated with 20 ng/mL EGF (B–D), and incubated at indicated times at 37°C. Arrows indicate membrane localization and arrowheads indicate vesicular localization. Single prime (ʻ) images represent single channel EGFR of inset, double prime (ʻʻ) images represent single channel MUC1 of inset, and triple prime (ʻʻʻ) images represent single channel EEA1 of inset. Scale bar represents 10 µm (A–D). Cells were incubated with either anti-EGFR 225 (green), anti-MUC1 Ab-5 (red), or anti-EEA1 H-300 (cyan) antibodies, and mounted with DAPI (blue). (**E**) Quantification of Pearson’s coefficient value r for EGFR-MUC1 co-association in black, EGFR-EEA1 co-association in grey. *n* ≥ 3 for all time points indicated. (**F**) Protein lysates were collected from BT20 cells (-S represents serum-starved; 5′, 30′, 60′ indicate time post-EGF treatment at 37°C incubation) and analyzed via immunoblot using the indicated antibodies. Molecular weights are indicated on the right.

We have previously demonstrated that MUC1 promotes EGFR-dependent breast cancer by inhibiting the degradation of EGFR and promoting its trafficking to the nucleus [18, 20]. To elucidate how MUC1 is altering trafficking, we investigated the effects of MUC1 expression on the canonical trafficking patterns of EGFR. We began by evaluating the colocalization of MUC1 and EGFR with EEA1, a marker of the early endosome in both BT20 and MDA-MB-468 breast cancer cells (Figure 1 and Supplementary Figure 1). To localize EGFR to the plasma membrane, cells were serum starved, where we observed plasma-membrane localized MUC1 and EGFR (Figure 1A, arrow). Upon treatment with 20 ng/mL EGF for 5 min, MUC1 and EGFR became colocalized with EEA1 in intracellular vesicles (Figure 1B, arrowheads). Unexpectedly, EGFR and MUC1 remained in EEA1-positive vesicles for at least 60 min treatment (Figure 1C and 1D, arrowheads, Figure 1E). Additionally, no loss of EGFR expression upon EGF treatment is observed in the presence of MUC1, as we have previously reported [18] (Figure 1F).

**Figure 2:**
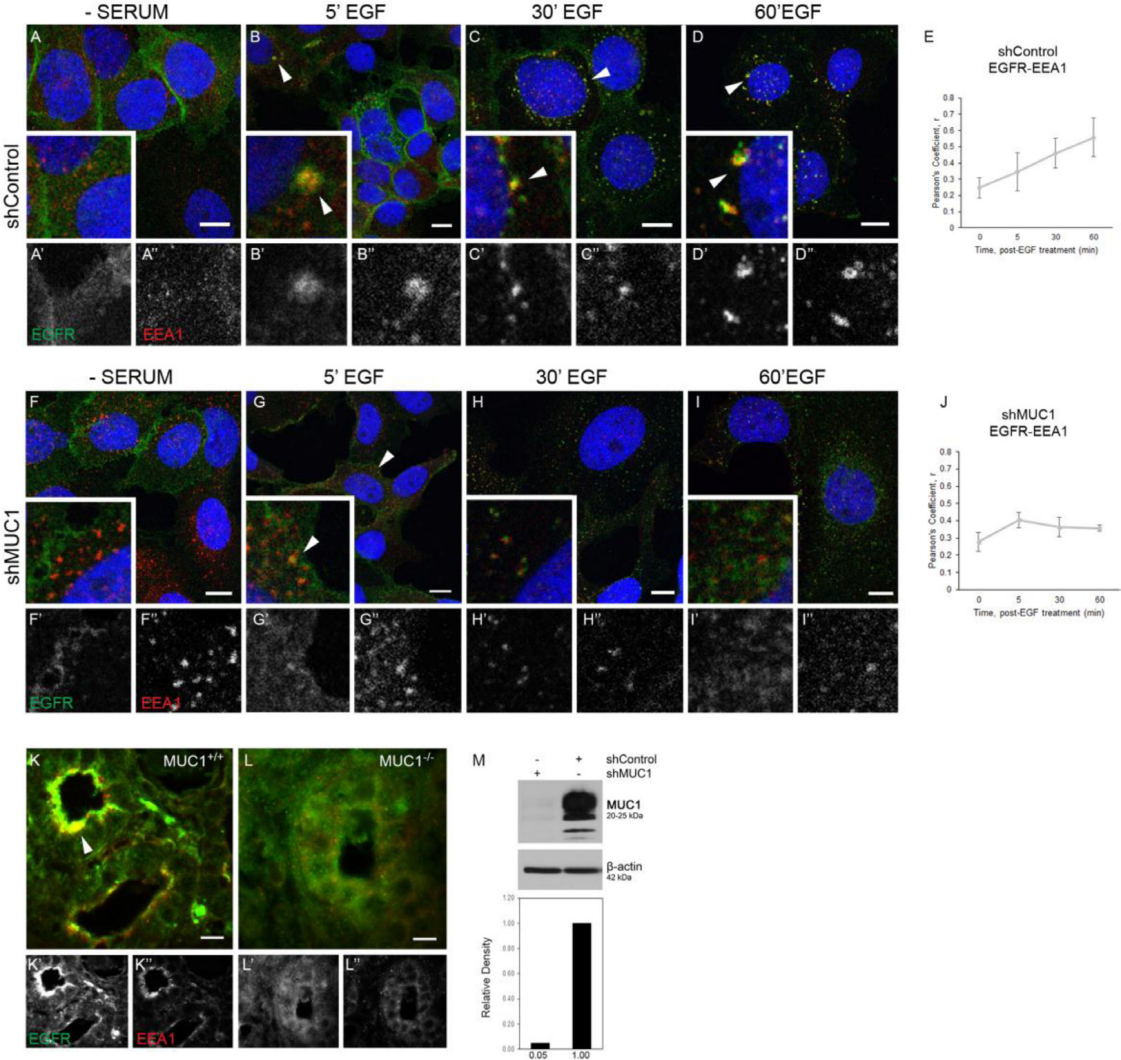
EGFR colocalization with EEA1 is prolonged and degradation is inhibited in the presence of MUC1 (**A–D**)**,** (**F–I**). BT20 cells were transfected with either control- or MUC1-specific shRNA (shControl, shMUC1 respectively). Cells were serum-starved and treated with 20 ng/mL EGF (B–D, G–I). Cells were incubated with either anti-EGFR 225 (green) or anti-EEA1 H-300 (red) and mounted with DAPI (blue). Arrowheads indicate vesicular localization. Single prime (‘) images represent single channel EGFR of inset, double prime (“) images represent single channel EEA1 of inset. (**E, J**) Quantification of Pearson’s coefficient value r for EGFR-EEA1 co-association. *n* ≥ 3 for all time points indicated. (**K, L**) Mammary glands taken from WAP-TGFα / MUC1^+/+^ (K) or WAP-TGFα / MUC1^–/–^ (L) were incubated with anti-EGFR 1005 G (green), anti-EEA1 H-300 (red). Representative images selected; *n =* 6. Colocalization highlighted by arrowhead. Scale bar represents 10 µm (A–D, F–I, K–L). (**M**) Protein lysates were collected from shRNA-treated BT20 cells and analyzed via immunoblot. Molecular weights are indicated on the right. Relative levels of MUC1 were quantified using ImageJ.

To determine if MUC1 was required for the prolonged retention of EGFR in EEA1-positive endosomes, BT20 cells were treated with either a non-specific control or MUC1-specific shRNA (previously optimized in [18, 20, 21]) and EGFR trafficking was followed via immunofluorescence. Cells treated with control shRNA (shControl; hereafter referred to as MUC1^+^ cells) behaved similarly as described in Figure 1, with EGFR restricted to the cell membrane in the absence of serum (Figure 2A), undergoing endocytosis to colocalize with EEA1 as early as 5 min (Figure 2B, arrowheads), and maintaining this colocalization throughout 60 min (Figure 2C and 2D, arrowheads, Figure 2E). In contrast, cells treated with the MUC1-specific shRNA (shMUC1; hereafter referred to as MUC1^–^ cells) demonstrated a significantly different phenotype. In these cells, EGFR colocalization with EEA1 was highest after 5 min post-EGF treatment (Figure 2G, arrowheads, Figure 2J), and then decreased to non-correlative levels (Figure 2H, 2I), as expected in EGFR trafficking to the lysosome for degradation [22] after initial localization at the cell surface post serum-starvation (Figure 2F). Verification of MUC1 knockdown is shown in Figure 2M, and experiments performed in MDA-MB-468 cells showed a similar phenotype (Supplementary Figure 2). As previously demonstrated, knockdown of MUC1 results in increased EGFR degradation upon EGF stimulation (Supplementary Figure 3). While we observed no changes in dual-phosphorylated ERK, we did observe an increase in phospho-AKT levels, a trend previously demonstrated to be associated with mislocalized EGFR and commonly found in cancers such as prostate, ovarian, and breast [18, 23, 24] (Supplementary Figure 3).

We had previously demonstrated that MUC1 expression drives EGFR-dependent breast cancer (in the WAP-TGFα transgenic mouse model), including >60% reduction of EGFR-driven tumor formation in the absence of MUC1 [19]. To determine whether EGFR colocalization with EEA1 was affected by MUC1 in this model, tumor sections were evaluated from either WAP-TGFα / MUC1^+/+^ or WAP-TGFα/MUC1^–/–^ mice [19]. In the presence of MUC1, EGFR was strongly apically localized with EEA1 (Figure 2K, arrowhead). In the absence of MUC1, little to no EEA1/EGFR colocalization was observed (Figure 2L) Together, these data demonstrate that MUC1 is promoting extended interactions between EGFR and EEA1-positive vesicles both *in vitro* and *in vivo*.

### MUC1 does not alter initial EGFR colocalization with EEA1, but does inhibit trafficking to the lysosome

**Figure 3:**
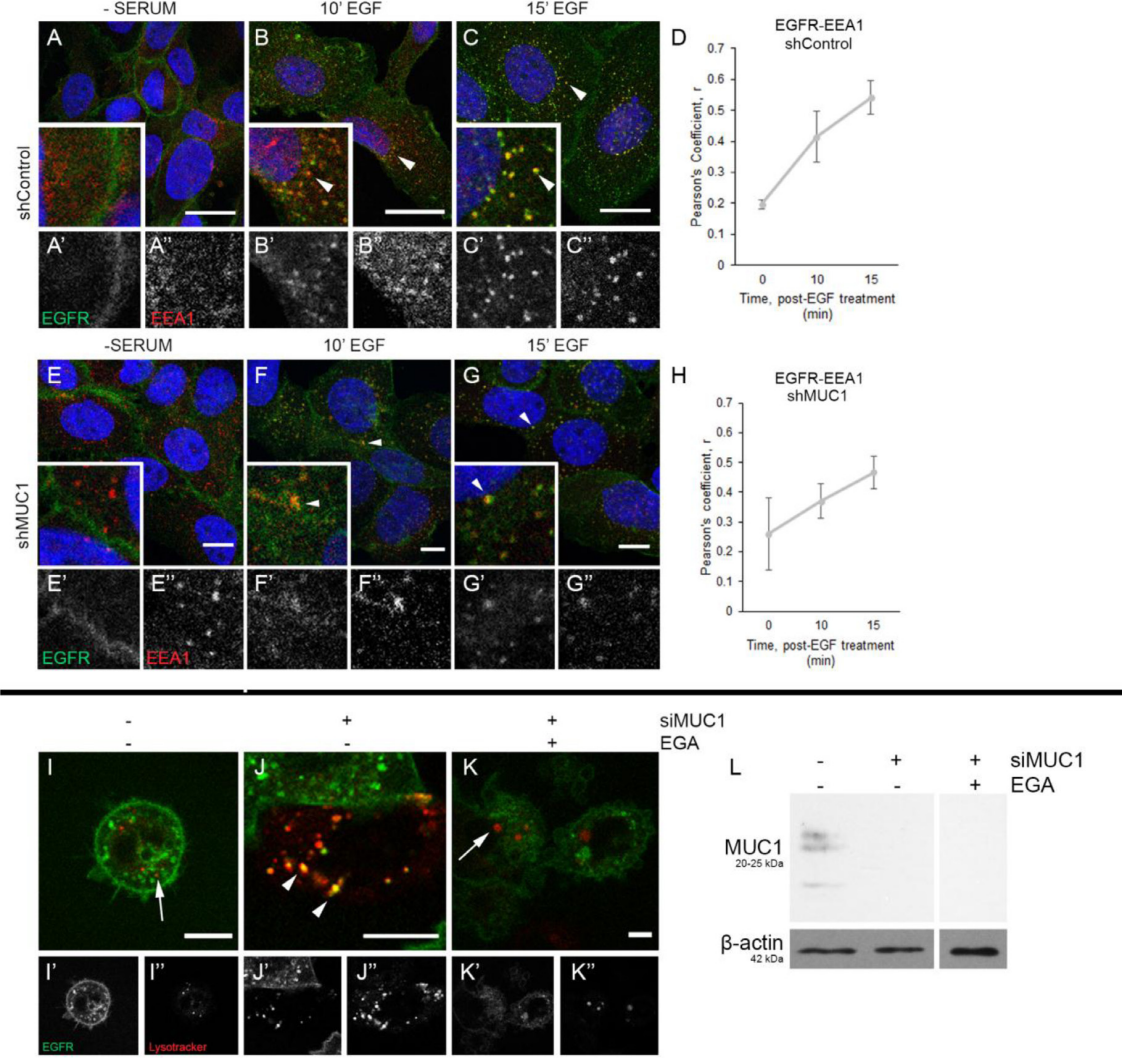
MUC1 does not delay EGFR association with EEA1, but does alter EGFR trafficking to the lysosome (**A**–**C**), (**E**–**G**) BT20 +/–MUC1 cells were generated as described in Figure 2 and analyzed. Cells were serum-starved overnight, treated with 20 ng/mL EGF (B–C, F–G), then evaluated. Cells were incubated with either anti-EGFR 225 (green) or anti-EEA1 H-300 (red) and mounted with DAPI (blue). Arrowheads indicate vesicular localization. Single prime (‘) images represent single channel EGFR of inset, double prime (“) images represent single channel EEA1 of inset. (**D, H**) Quantification of Pearson’s coefficient value r for EGFR-EEA1 co-association. *n =* 3 for all time points indicated. (**I–K**) MDA-MB-468 cells were transfected with EGFR-GFP and transduced with MUC1-specific siRNA (J–K). Cells were incubated with Lysotracker Red, followed by 10 min treatment with EGF, prior to incubation with DMSO (I–J) or 20 µM EGA (K). Single prime (‘) images represent single channel EGFR (green), double prime (“) images represent single channel Lysotracker (red). Arrows indicate lysosomes, arrowheads indicate vesicular colocalization. Scale bar represents 10 µm (A–C, E–G, I–K). Protein lysates were made upon completion of imaging and 20 µg were separated by SDS-PAGE. (**L**) Relative levels of proteins were determined as shown. Molecular weights are indicated on the left.

To elucidate the stage at which MUC1 alters EGFR endocytosis, cells were exposed to EGF and incubated for 10-15 min, and immunofluorescence was performed. In both the presence or absence of MUC1, we observed EGFR accumulation at the plasma membrane after serum-starvation without EEA1 colocalization (Figure 3A, 3E). After 10 min treatment of EGF, EGFR is internalized and is associated with EEA1-positive vesicles (Figure 3B and 3F, arrowheads). This correlation increases through 15 min (Figure 3D, 3H), with EGFR and EEA1 colocalizing in vesicles throughout the cytoplasm, regardless of MUC1 expression (Figure 3C and 3G, arrowheads). This indicates that MUC1 does not affect the rate at which EGFR progresses from the plasma membrane into EEA1-positive vesicles.

We next evaluated whether MUC1 alters the trafficking of EGFR to the lysosome. Using MDA-MB-468 cells, EGFR localization to the lysosome was visualized using an EGFR-GFP fusion protein and Lysotracker Red during live imaging assays. In MUC1^+^ cells, EGFR fails to localize to the lysosome, even after 90 min of EGF treatment (Figure 3I, arrow). In contrast, EGFR in MUC1^-^ cells can be found associated with the lysosome (Figure 3J, arrowheads; Supplementary Video 1). Verification of MUC1 knockdown is shown in Figure 3L. To determine if trafficking to the lysosome can be ablated independently of MUC1 knockdown in these cells, cells were treated with the late endosome trafficking inhibitor EGA (4-bromobenzaldehyde *N*-(2,6-dimethylphenyl)semi-carbazone) [25]. This led to the sequestration of EGFR to intracellular vesicles similar to what is observed in MUC1^+^ cells (Figure 3K). We next investigated whether MUC1 may be promoting the mislocalization of EGFR to either the golgi or mitochondria, as has previously been demonstrated [11, 14]. Upon evaluation we found that EGFR failed to colocalize with the trans-golgi apparatus visible by antibody TGN-46. We also failed to see EGFR colocalization with the mitochondrial marker COX IV (Supplementary Figure 4).

### Intracellular pools of EGFR increase migration of breast cancer cells

**Figure 4:**
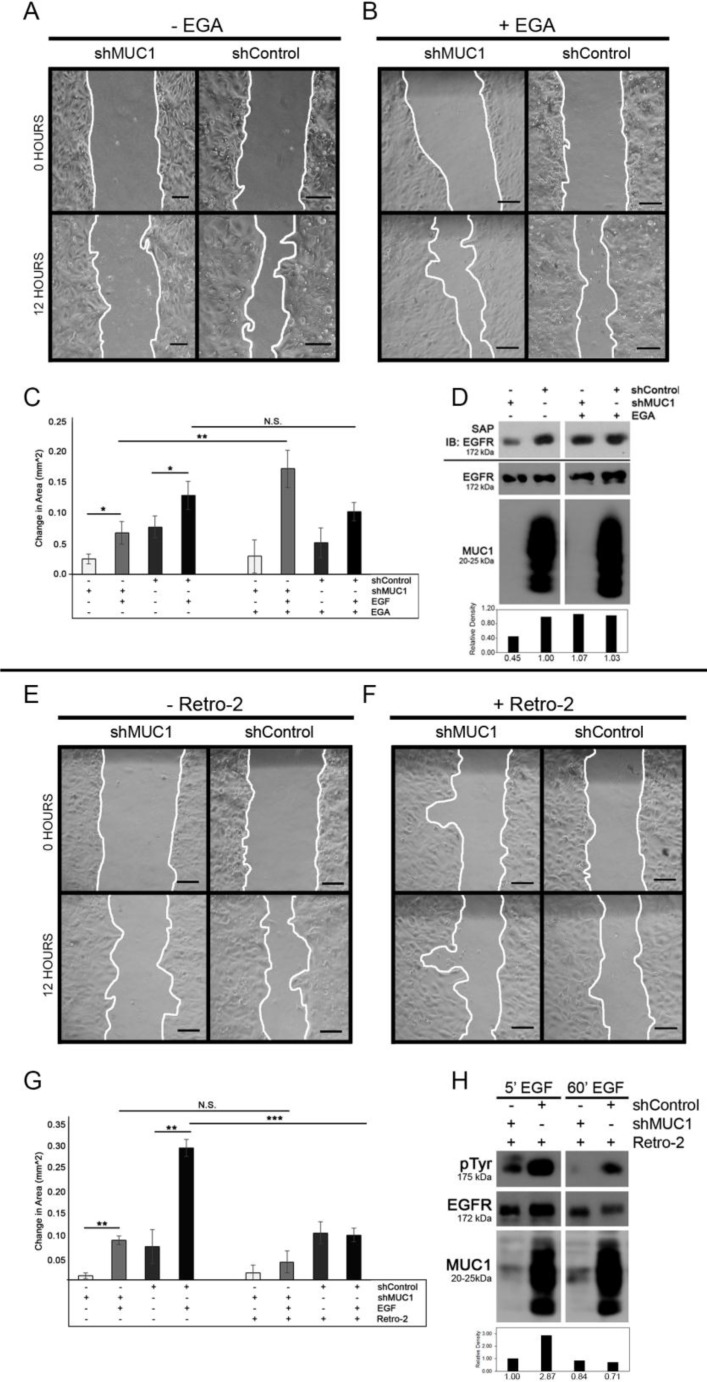
MUC1 promotes EGF-dependent migration through retrograde trafficking BT20 +/–MUC1 cells were generated as described in Figure 2 and analyzed (A–H). (**A–C**) Cells were grown to confluence, serum-starved and scratched in PBS, then observed for migration in serum-free media with either the absence (C) or presence of 20 ng/mL EGF (A–C). Cells were also treated with either DMSO (A, C) or 20 µM EGA (B, C). Migration was determined by measuring the area of the wound as indicated by the white lines (A, B) and quantifying the difference after 12 hours (C). Scale bar represents 10 µm. *n =* 3. Error bars show ± standard deviation. ^*^p < 0.05, ^**^p < 0.01. (**D**) Cells were treated with DMSO or EGA for 15 min, then biotinylated and treated with EGF and EGA. Lysates were taken and immunoprecipitated using streptavidin agarose and separated by SDS-PAGE. SAP = streptavidin agarose pulldown, immunoblotted as shown. Molecular weights are indicated on the left. Relative levels of surface EGFR were quantified using ImageJ. (**E–G**) Cells were plated for a wound healing assay and observed for migration in serum-free media with either the absence (G) or presence of 20 ng/mL EGF (E–G). Cells were treated with either DMSO (E, G) or 50 µM Retro-2 (F, G). *n =* 3. Error bars show ± standard deviation. ^**^p < 0.01, ^***^p < 0.001. (**H**) Cells were treated with DMSO or Retro-2 for 60 min, then treated with EGF and Retro-2. Lysates were taken and separated by SDS-PAGE. Molecular weights are indicated on the left. Relative levels of total EGFR were quantified using ImageJ.

MUC1 co-expression with EGFR can promote cell migration and metastasis [19, 21, 26]. To investigate whether intracellular pools of EGFR-MUC1-EEA1 vesicles were associated with increased migratory potential, BT20 cells were evaluated by a wound-healing assay in the presence or absence of MUC1. After exposure to EGF under serum-starved conditions, both MUC1^+^ and MUC1^-^ cells showed a significant increase in migration, with MUC1^+^ cells migrating significantly larger distances after 12 hours (Figure 4A; quantified in 4C). To determine the effect of MUC1 on driving migration by inhibiting EGFR trafficking to the lysosome, cells were treated with the late endosome trafficking inhibitor EGA in the presence or absence of EGF. We first verified that EGA blocked lysosomal degradation of EGFR by biotinylating surface EGFR and internalizing by EGF stimulation. We found that EGA treatment inhibited EGF-dependent EGFR degradation to a similar extent as MUC1 expression (Figure 4D). We next treated cells with EGA and evaluated effects on migration. While blocking EGF-dependent EGFR trafficking to the lysosome resulted in increased migration in MUC1^-^ cells (142% increase in area, Figure 4B; 4C), this did not occur in MUC1^+^ cells. This indicates that MUC1^+^ cells have already blocked lysosomal transfer of EGFR, making EGA treatment irrelevant. Together, these data indicate that MUC1 is promoting EGF-dependent migration by promoting alternative trafficking of EGFR, and that plasma membrane localized EGFR does not drive the pro-migratory phenotype.

Since MUC1 is redirecting EGFR away from the lysosome to be retained in EEA1-positive vesicles that are localized primarily in the perinuclear space, we next investigated if the migratory phenotype was dependent upon retrograde trafficking. To investigate retrograde trafficking of EGFR, MUC1^+^ or MUC1^-^ cells were treated with the retrograde trafficking inhibitor Retro-2 [27]. MUC1^+^ cells treated with Retro-2 and EGF demonstrated a significant decrease (66% reduction in area) in migratory ability compared to those treated with DMSO (Figure 4F; 4E, respectively) while MUC1^-^ cells were not significantly affected by the addition of Retro-2 (Figures 4F; 4G). However, when cells were treated with Retro-2 in the absence of serum or ligand, MUC1 expression has little effect on migration (Figure 4G), indicating that MUC1-driven migration is dependent upon EGFR undergoing retrotranslocation. Changes seen in migration of MUC1^+^ cells were not driven by changes in proliferation, regardless of EGF presence (Supplementary Figure 5). EGFR degradation was verified by treating cells with EGF and Retro-2 and measuring EGFR phosphorylation and expression via western blot (Figure 4H).

### MUC1-driven retrograde trafficking is required for EGFR-dependent cytoskeletal rearrangement and migration

**Figure 5:**
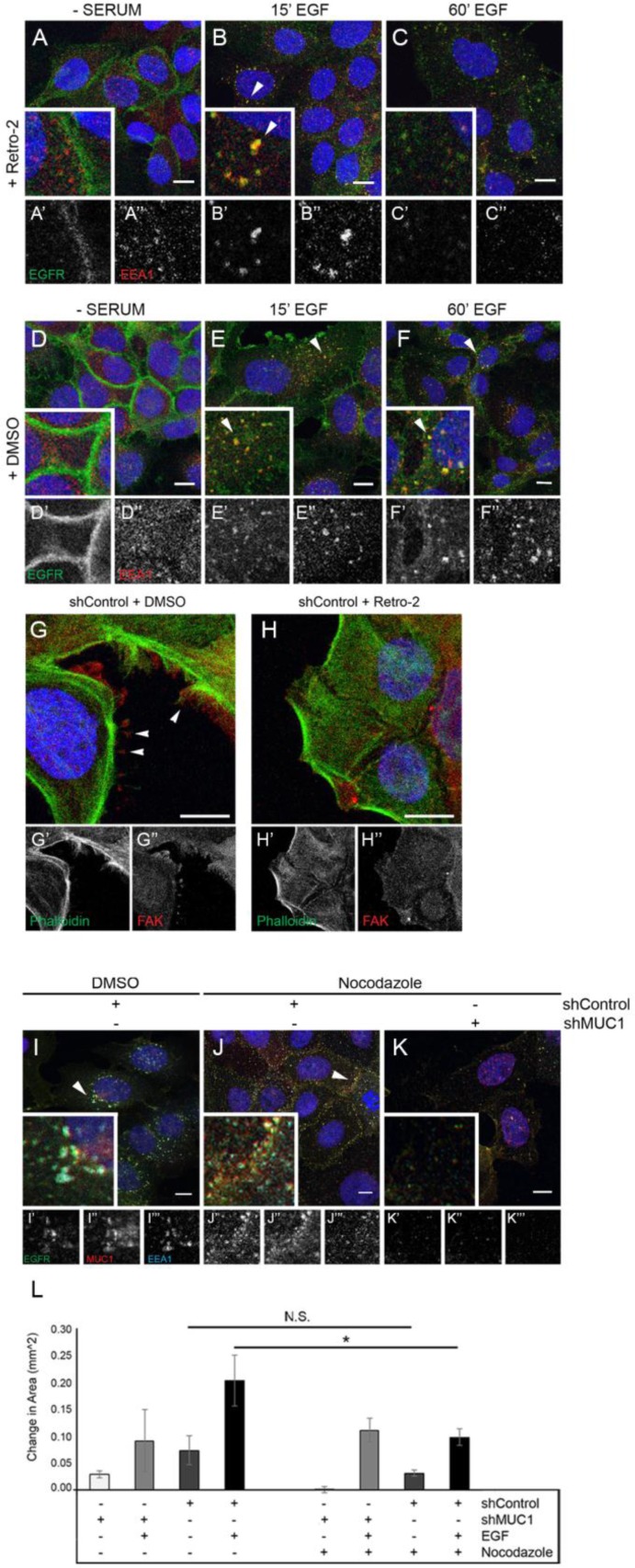
Inhibition of retrograde trafficking promotes EGFR degradation and cytoskeletal rearrangement resulting in a loss of migratory ability BT20 MUC1^+^ cells were generated as described in Figure 2. (**A–F**) Cells were serum-starved overnight, treated with DMSO or 50 µM Retro-2 followed by 20 ng/mL EGF (B, C, E, F), then incubated with DMSO or 50 µM Retro-2. Cells were incubated with either anti-EGFR 225 (green) or anti-EEA1 H-300 (red) and mounted with DAPI (blue). Arrowheads indicate vesicular localization. Single prime (ʻ) images represent single channel EGFR of inset, double prime (ʻʻ) images represent single channel EEA1 of inset. (**G–H**) Cells were serum-starved, treated with either DMSO or 50 µM Retro-2 for 60 min, followed by 20 ng/mL EGF, then incubated with DMSO or 50 µM Retro-2 for 60 min. Cells were incubated with FAK A-17 (red) and phalloidin-488 (green) and mounted with DAPI (blue). Arrowheads indicate FAKs. Single prime (ʻ) images represent single channel phalloidin, double prime (ʻʼ) images represent single channel FAK. (**I–K** ) BT20 +/– MUC1 cells were serum-starved and treated with 20 ng/mL EGF for 60 min along with either 1 µM nocodazole or DMSO. Arrowheads highlight vesicular EGFR localization. Scale bar represent 10 µm (A–K). (**L**) shRNA-treated BT20s were prepared for wound healing assays, then observed for migration in serum-free media with either the absence or presence of 20 ng/mL EGF. Cells were also treated with either DMSO or 1 µM nocodazole. *n =* 3. Error bars show ± standard deviation. ^*^p < 0.05.

To further evaluate the MUC1-dependent retrograde trafficking of EGFR, we evaluated how Retro-2 treatment would impact the localization of EGFR and EEA1. In the absence of EGF, EGFR is localized to the plasma membrane in the presence or absence of Retro-2 (Figure 5A and 5D). In cells treated with DMSO, EGFR enters and remains in EEA1-positive intracellular vesicles upon EGF treatment as previously shown (Figure 5B; Figure 2C–2D). In contrast, when retrograde trafficking is blocked, EGFR and EEA1 colocalization is lost in a MUC1-dependent manner (Figure 5C). EGFR colocalization with EEA1 is unaffected by Retro-2 in the absence of MUC1 (data not shown). These data indicate that the EGFR-EEA1 vesicles are trafficking via retrotranslocation when in the presence of MUC1.

As retrotranslocation is required for MUC1-dependent migration in response to EGF, we next evaluated whether retrotranslocation was affecting cell phenotype. In these experiments, cells were treated with Retro-2 in the presence or absence of MUC1, while colocalizing EGF-Alexa 647 with Phalloidin-Alexa 488. Without EGF treatment, actin organization was unaffected by the presence or absence of MUC1 (Supplementary Figure 6A–6B, 6E–6F). In contrast, when exposed to EGF, MUC1^+^ cells display frequent membrane extensions which are not seen in MUC1^-^ cells (Supplementary Figure 6C) and these revert to diffusely distributed actin when MUC1^+^ cells are treated with Retro-2 (Supplementary Figure 6D). Note that no change was observed in MUC1^-^ cells when treated with EGF in the absence or presence of Retro-2 (Supplementary Figure 6G; 6H). To determine the identity of these protrusions, cells were evaluated for the formation of FAK-positive structures (Focal Adhesion Kinase), which can drive migration [28, 29]. Structures consistent with focal adhesions were apparent in MUC1^+^ cells after exposure to 60 min of EGF (Figure 5G, arrowheads), a phenotype that is lost when Retro-2 is introduced (Figure 5H). Together, these results indicate that MUC1 is promoting retrograde trafficking of EGFR that results in the formation of FAK-positive membrane protrusions.

Previous studies have demonstrated that interactions between EGFR and microtubules are known to promote trafficking of EGFR to intracellular structures [30]. To elucidate the effects of MUC1 expression on the interaction between EGFR and the cytoskeleton, we evaluated the localization of EGFR upon the addition of EGF and nocodazole (an inhibitor of microtubule polymerization). After 60 min, while nocodazole does not block the colocalization of EGFR, MUC1, and EEA1, it does alter their location within the cell. The addition of nocodazole re-localizes EGFR, MUC1, and EEA1-containing vesicles from the perinuclear space (Figure 5I, arrowhead), to the subapical region of the cell surface (Figure 5J, arrowhead). Nocodazole does not appear to effect MUC1^-^ cells (Figure 5K). These data indicate that MUC1 may be blocking the association between EGFR and actin and re-localizing it (via microtubules) to perinuclear locations.

Finally, to determine if the migration associated with retrograde trafficking would be affected by blocking interactions between EGFR and microtubules, cells were treated with nocodazole. Upon addition of nocodazole, MUC1^+^ cells demonstrated a significant decrease in migratory capacity (50% reduction in area) when simultaneously exposed to 20 ng/mL EGF (Figure 5L). This trend was not observed in MUC1^-^ cells, which migrated relatively equal distances in the presence of EGF and nocodazole (Figure 5L). This decrease in migration was not due to cell death, confirmed by three-day cell survival assays (data not shown). Together, these data indicate that the retrograde trafficking driven by MUC1 promotes a microtubule-dependent re-localization of EGFR that drives migration.

### Intravesicular EGFR decreases the efficacy of extracellular-domain-targeted therapeutics

**Figure 6:**
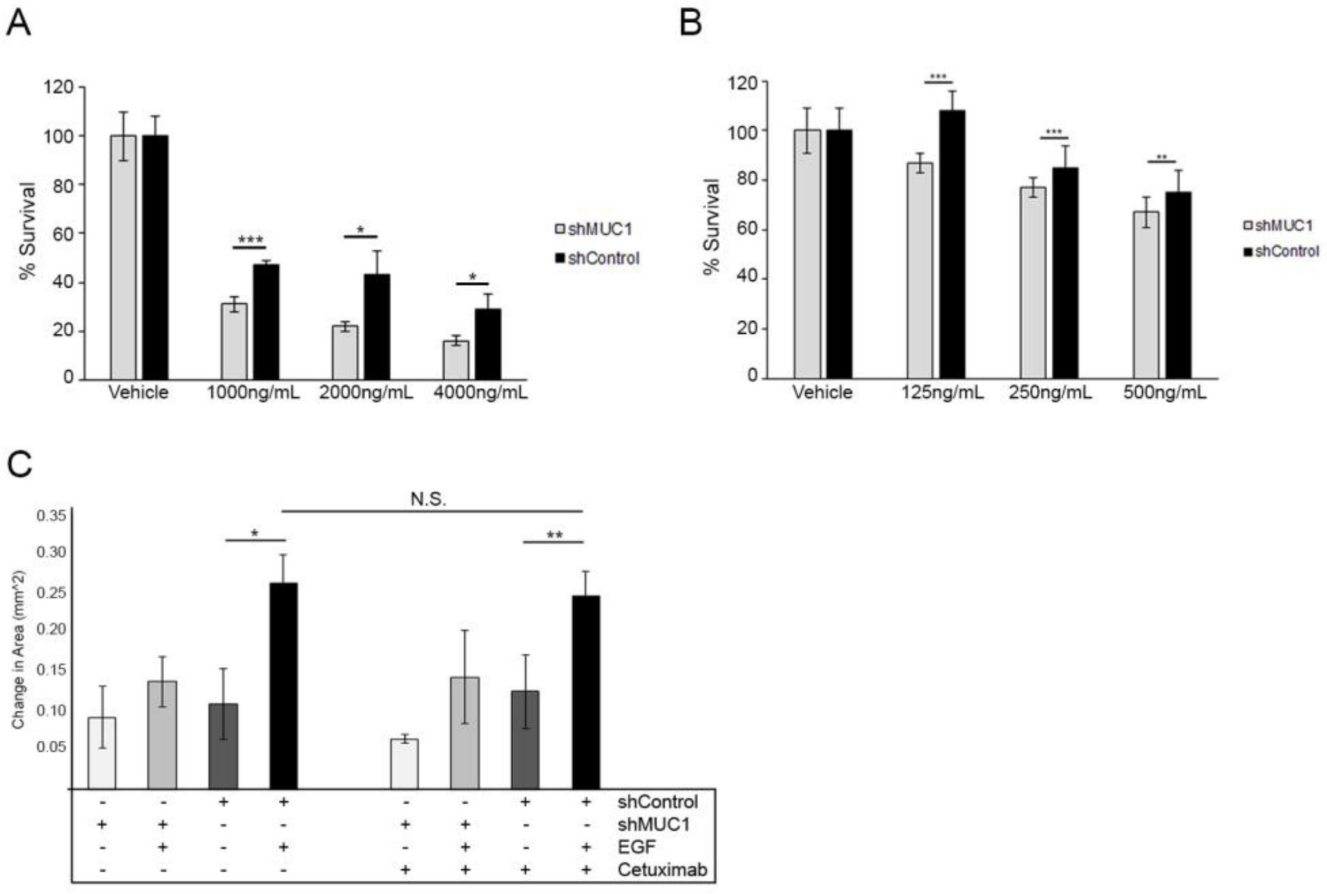
Altered localization of EGFR decreases the efficacy of Cetuximab (**A**) Cell viability assay performed in BT20 cells over 3 days comparing treatment with Cetuximab to MUC1^+^ cells (black) to MUC1^–^ cells (grey). Data shown represents mean +/– percent difference of assays performed in triplicate. ^*^p < 0.05, ^***^p < 0.001. (**B**) Cell viability assay performed in MDA-MB-468 cells comparing treatment with Cetuximab to MUC1^+^ cells (black) to MUC1^–^ cells (grey). Data shown represents mean +/– percent difference of assays performed in triplicate. ^**^p < 0.01, ^***^p < 0.001. (**C**) Cells were plated for a wound healing assay as described in Figure 5 and observed for migration in serum-free media in either the absence or presence of 20 ng/mL EGF. Cells were treated with 0.1% DMSO or 100 ng/mL Cetuximab. Wound closure was determined by measuring the area of the wound and quantifying the difference after 12 hours. *n =* 3. Error bars show ± standard deviation. ^*^p < 0.05, ^**^p < 0.01.

Given the apparent role of intravesicular EGFR in driving migration, we next investigated the effects of MUC1 expression on the activity of Cetuximab in cell survival, a monoclonal antibody directed at the extracellular domain of EGFR, responsible for inhibiting ligand binding and receptor dimerization, while promoting receptor internalization and degradation [31–35]. We observed a significant reduction in cell survival in MUC1^–^ versus MUC1^+^ cells after 72 hours of Cetuximab treatment (Figure 6A; 6B), indicating the effects of EGFR internalization on the effectiveness of Cetuximab. Concentrations of Cetuximab varied between cell lines, as MDA-MB-468 cells display EGFR amplification [36, 37]. Note that while MUC1 promotes the retention of intracellular EGFR, it can also promote surface recycling of EGFR over time [18]. We also demonstrated no significant change in migration ability of cells when treated at relatively low levels of Cetuximab (levels that resulted in a 95% +/– 3% survival after 24 hours treatment, data not shown) in MUC1^+^ or MUC1^-^ cells (Figure 6C), indicating that it is not surface EGFR responsible for driving migration. Taken together, retrograde trafficking of EGFR is responsible for driving migration in triple negative breast cancer, and its altered localization inhibits the effectiveness of the anti-EGFR antibody Cetuximab.

## DISCUSSION

In this study, we demonstrate that MUC1 expression alters EGFR trafficking by promoting retention in EEA1-positive vesicles while limiting trafficking to the lysosome. These events are driven by association with MUC1 at the plasma membrane, which promotes its microtubule-dependent intracellular localization. EGFR colocalized in EEA1-positive vesicles undergoes retrograde trafficking that results in membrane protrusion and migration, reducing efficacy of an anti-EGFR antibody.

It was previously shown that apically localized EGFR is significantly less efficient at downregulation compared to its localization to the basolateral domain [38], an effect that is recapitulated by EGFR-MUC1 colocalization. MUC1 is a highly glycosylated transmembrane mucin restricted to the apical surface of ductal epithelial cells. It is involved in signal transduction, inhibition of cell-cell adhesion, and promotion of cell migration [39–41]. In normal tissue, MUC1 undergoes continual endocytosis and recycling to maintain maximal levels of O-linked glycosylation [42]. MUC1 endocytosis is known to be clathrin-mediated, as well as dynamin-dependent [43]. Vesicular MUC1 has been shown to require the GTPase Rab5 for fusion with early endosomes, but will not accumulate in sorting endosomes under steady-state conditions [43, 44]. The Rab5 effector EEA1 is also essential for EGFR trafficking, as EEA1 mediates endosome docking and promotes vesicular fusion [45, 46]. Our present study indicates that the association of MUC1 with EGFR does not alter the entrance into EEA1 vesicles, but does promote their retention. Recently, Murray *et al.* demonstrated that EEA1 works to capture vesicles for fusion when in an extended confirmation, an inducible event to change from a flexible state [47]. Given the association of EGFR with MUC1 within intracellular vesicles, it is possible that MUC1 is acting as a conformational inhibitor to EEA1 and altering its transformation into the necessary extended conformation.

We have previously demonstrated that MUC1-EGFR complexes are capable of localizing around and entering the nucleus, acting as a co-transcriptional activator of genes upregulated in breast cancer, such as cyclin D1 [20]. It has also been shown that EGFR and MUC1 will accumulate in the perinuclear space in pancreatic cancer cells after exposure to EGF stimuli [48], similar to our current findings (Figure 1E–1F, Figure 2C–2D). Under oxidative stress, EGFR is capable of being trafficked to the perinuclear space in a non-clathrin mediated mechanism resulting in constitutive activation of the receptor [49]. EGFR has also been found to accumulate in the perinuclear space of cells in non-small cell lung cancer cells, but only under conditions in which EGFR has been mutated, allowing for increased interactions between EGFR and the cancer-associated protein Src [50], emphasizing the abnormality of extended EGFR retention in the perinuclear space.

Retro-2 is a small molecule inhibitor that works to inhibit retrograde trafficking between early endosomes and the trans-golgi network, designed against ribosome-inactivating proteins such as Shiga-like toxins and ricin [27, 51]. Unlike other retrograde inhibitors, it does not affect cell viability or compartment morphology [27, 52]. It also has been demonstrated to affect neither recycling of the transferrin receptor nor lysosomal degradation of EGFR, allowing us to study retrograde trafficking of EGFR without altering other canonical pathways. Interestingly, our results do not show EGFR/EEA1/MUC1 vesicles joining the Trans Golgi Network, but instead being maintained as discrete vesicles within the cell over time. Furthermore, this localization is strongly associated with EGF-dependent migration that is lost upon the breakdown of microtubules.

Filopodia have been observed extensively in metastatic cells and can drive migration and metastasis (reviewed in [53]). Some studies have shown that upregulated FAK, which is found in filopodia, are also associated with more than 80% of primary breast cancer sites and 100% of metastatic sites [54, 55]. While these studies correlate filopodia-like formation and EGFR retrotranslocation from the cell surface to sustained EGFR/EEA1/MUC1 positive vesicles, future studies will be performed to determine the mechanism by which these events are linked.

Our data indicate that MUC1 alters EGFR trafficking by promoting retention in EEA1-positive vesicles and limiting downregulation via the lysosome. MUC1 may drive the oncogenic potential of cells by generating and sustaining intracellular pools of activated EGFR, leading to an increase in migratory events. Current studies have demonstrated that intracellularly-localized EGFR is associated with poor prognosis, increased malignant potential, and decreased disease-free survival rates of patients with squamous cell and ovarian carcinomas [26, 56, 57]. Monoclonal antibody treatments such as Cetuximab are therapies directed at the extracellular domain of EGFR, designed to inhibit ligand binding and dimerization [33, 58, 59]. Our data point to a mechanistic role for internalized EGFR and highlights the fact that a functional pool of EGFR does not reside on the cell surface, which may be inhibiting the effectiveness of anti-EGFR therapeutics.

## MATERIALS AND METHODS

### Cell culture

BT20 breast cancer cells were obtained from ATCC and cultured in Dulbecco’s modified Eagle’s medium (Corning), 10% Fetal Bovine Serum (Peak Serum Inc), and 1% Pen/Strep (Corning). MDA-MB-468 breast cancer cells were obtained from ATCC and cultured in RPMI-1640 medium (Corning), 5% FBS (Peak), and 1% Pen/Strep (Corning). Cells were tested for mycoplasma through the Arizona Cancer Center Experimental Mouse Shared Resource.

### shRNA and siRNA transduction

BT20 and MDA-MB-468 cells were transduced with either a lentiviral control or MUC1-specific shRNA or siRNA construct. Particles were purchased from Sigma-Aldrich using sequences described previously [18]. For shRNA transduction, briefly, 30 000 cells were plated in a 24-well dish and 24 hours later treated with lentiviral particles containing a CFP-tag with an MOI of 2. Cells were left untreated for 48 hours, followed by selection with 0.2–0.3 µg/mL puromycin (Fisher Scientific) until only CFP-expressing cells remained. For siRNA transduction, 100 000 cells were plated in 60mm dishes and 24 hours later transfected with control or MUC1 siRNA (sequence previously described [18]) at a concentration of 4.8 nmol using the manufacturer’s protocol for Lipofectamine RNAiMAX (Invitrogen). Experiments were performed after 48 hours of siRNA exposure.

### Immunofluorescence

#### Cell culture

30 000 cells were plated in plastic 8-chamber slides (Falcon) and allowed to reach 90% confluency. Cells were serum-starved 16 hours, treated for 10 min on ice with 20 ng/mL EGF (Corning)-containing SF MEM or RPMI, washed with PBS, and then incubated for 5–120 min at 37°C in SF MEM or RPMI. Cells were fixed with 4% PFA (Santa Cruz) for 5 min, then permeabilized with 0.5%Triton-X 100/0.05% NaN3/PBS for 15 min. Cells were blocked for 60 min in 20% FBS/0.05% NaN3, followed by incubation with primary antibodies in 10% FBS/0.05% NaN3 overnight at 4°C. Cells were washed 3X in 10% FBS/0.05% NaN3 (washing buffer), followed by 60 min treatment with secondary antibodies in washing buffer at RT in the dark. Cells were washed 6X in washing buffer, then treated with ProLong Diamond Antifade Mountant with DAPI (Molecular Probes). Slides incubated O/N at RT, before being imaged or stored at 4°C.

#### Phalloidin staining

Cells were treated with either 0.2% DMSO or 50 µM Retro-2 for 60 min at 37°C in SF MEM, followed by new SF MEM with either DMSO or Retro-2 with 20 ng/mL EGF-647 (EGF biotinylated complexed with 647-streptavidin, Molecular Probes) for 60 min at 37°C. Cells underwent quick fixation with Alexa-Fluor 488 Phalloidin (Molecular Probes) in 4% PFA for 20 min at 4°C in the dark. Cells were washed 3X in 1X PBS and treated with ProLong Antifade with DAPI (Molecular Probes).

#### Mammary glands

Glands were taken as previously described [19]. Glands were deparaffinized in xylenes, followed by EtOH/PBS hydration. Glands were treated for 3 min with Fc Receptor blocker (Innovex Biosciences), then incubated 20 min at RT in 3% BSA/PBS. Glands were incubated with Cyto Q HRP Enhancing Wash Buffer (Innovex Biosciences) for 30 seconds, then incubated with primary antibodies in blocking buffer O/N at 4°C. Glands were washed 5 × 30 sec in Enhancing Wash Buffer, then incubated in secondary antibodies in PBS O/N at 4°C. Glands were washed 5 × 30 sec in Enhancing Wash Buffer before being mounted with ProLong Diamond Antifade. (Molecular Probes). All animals were maintained as outlined by University of Arizona Institutional Animal Care and Use Committee (IACUC).

#### Antibodies

For cell culture, primary and secondary antibodies were used at 1:300 concentrations. EGFR clone 225 (Milipore). MUC-1 Ab-5 (Thermo Fisher Scientific). EEA1 H-300 (Santa Cruz Biotechnology). COX-IV (Cell Signaling 3E11). TGN46 (Sigma 7576). FAK A-17 (Santa Cruz) was used at a 1:50 primary and 1:100 secondary concentration. For mammary glands, primary antibodies were used at 1:300 concentrations; secondary antibodies were used at 1:500 concentrations. EGFR 1005-G (Santa Cruz Biotechnology). AlexaFluor 488 donkey anti-mouse IgG (Invitrogen), AlexaFluor 594 donkey anti-rabbit IgG (Invitrogen), AlexaFluor 594 donkey anti-goat IgG (Invitrogen), AlexaFluor 647 goat anti-armenian hamster IgG (Jackson ImmunoResearch).

#### Imaging

Cell culture images were taken at 63X using a Leica SP5-II confocal microscope, courtesy of the Imaging Shared Resource at the Arizona Cancer Center. Tissue images were taken using a Leica DMLB microscope and Leica DFC 310 FX camera mounted on a 1x C-mount using the LAS V4.5 software. Co-association was quantified using JACoP plugin for ImageJ to determine Pearson’s coefficients.

### Western blots

Cells were lysed in ice-cold cell lysis buffer containing 20 mM Tris pH 7.4, 150 mM NaCl, 1% NP40, 5 mM EDTA pH 8.0, 1% NaF, 1% NaVO4, 0.1% NH4Molybate, and 8% complete phosphatase and protease inhibitors (Roche). Lysates were sonicated 3X15 seconds at 15amplitude using a QSonica Q55 Sonicator before being centrifuged for 3 minutes at 13 200 rpm at 4°C. Supernatants were collected and stored at –80°C. Lysates were separated by SDS-PAGE on 5–20% or 10% Acrylamide (Fisher Scientific) gels and transferred to 0.22 µm PVDF membranes (Milipore). Membranes were blocked in 3% BSA/TBS/0.1% Tween-20 EGFR, β-actin, dpERK, pAKT antibodies. Membranes were blocked in 3% milk/PBS/0.1% Tween-20 for EEA1; 1% milk/PBS/0.1% Tween-20 for AKT, MUC1. Bands were visualized using SuperSignal West Pico Chemiluminescent Substrate (Thermo Fisher Scientific).

#### Antibodies

EEA1 (Abcam ab137403). β-actin (Sigma A5441). dpERK (Sigma M8159). pAKT T308 (Cell Signaling 9275). AKT (Cell Signaling 9272). Secondary antibodies included HRP-Rabbit Anti-Goat IgG (Invitrogen), HRP-Goat Anti-Mouse IgG (Invitrogen), HRP-Goat Anti-Rabbit IgG (Life Technologies), HRP-Goat Anti-Armenian Hamster (Jackson ImmunoResearch).

### Immunoprecipitation assays

Lysates were taken as previously described under western blots. A working buffer of TNEN (50 mM Tris, 150 mM NaCl, 1 mM EDTA pH 8.0, 0.5% NP40) + inhibitors (10 mM NaF, 2 mM NaVO4, 50 µM NH4Molybate, 4% complete), 0.75–1 mg of protein, and 1.2 µg/mL EGFR Ab-13 (NeoMarkers) were immunoprecipitated and then loaded into an acrylamide gel for SDS-PAGE.

### Biotinylation assays

BT20 cells were serum-starved up to 16 hours prior. Cells were washed with cold 1X PBS, then treated with 1X biotin (EZ-Link Sulfo-NHS-SS Biotin) (Thermo Fisher Scientific) in PBS for 30 min at 4°C. Biotinylation reaction was quenched by washing 3X with Tris Quenching Buffer (10 mM Tris pH 7.4, 154 mM NaCl). Cells were then treated with 20 ng/mL EGF in SF MEM for 10 min on ice, washed 2X with 1X PBS, and incubated at 37°C for 5-120 min in SF MEM. Lysates were taken as previously described and immunoprecipitated.

### Lysotracker/Live imaging

MDA-MB-468 cells were transfected with an EGFR-GFP construct (a kind gift from G. Carpenter) and maintained with 0.5 mg/mL G418(Geneticin) (Life Technologies) selection. Cells were treated with 150 nM Lysotracker DND-99 (Molecular Probes) for 80 min at 37°C. Lysotracker-containing media was removed and cells were treated with SF RPMI + 20 ng/mL EGF and imaged every 5 min for up to 120 min using the Leica SP5-II confocal microscope at 63X at 37°C.

### Wound healing assays

50–100 000 cells were plated in 24-well dishes and allowed to reach 95% confluency. Cells were serum-starved, then covered in PBS and scratched with a p200 pipette tip. SF media with or without EGF (20 ng/mL), and with or without inhibitors (20 µM EGA (Milipore), 50 µM Retro-2 (1085, Sigma), 1 µM Nocodazole (1665, Sigma), 100 ng/mL Cetuximab (Milipore), or inhibitor controls – 0.1–0.2% DMSO) was added onto cells, and imaged every 4 hours. Images were quantified using ImageJ to determine the difference in area of the scratch against time 0 distance. *n =* 3 for each experimental group. Each experiment was repeated with at least 3 biological replicates using different cell lines and transductions. Graphical representation used the mean as the center value with error bars representing on standard deviation in each direction. *P*-values were calculated from a one-way ANOVA.

### Cell viability assay

2 000 cells were plated in 96-well dishes and allowed to grow for 2 days. Cells were then treated with various concentrations of Cetuximab or Retro-2 for 72 hours. Percent survival was calculated by comparing the treated wells to relative vehicle-treated cells.

## SUPPLEMENTARY MATERIALS FIGURES AND VIDEO




