# Supplementary material for: Intravesicular epidermal growth factor receptor subject to retrograde trafficking drives epidermal growth factor-dependent migration
Source: Oncotarget. 2017 Dec 29;9(5):6463–77. doi: 10.18632/oncotarget.23766 (PMC5814225; doi:10.18632/oncotarget.23766)
Supplement: Supplementary file 1 [file oncotarget-09-6463-s001.pdf]

Intravesicular epidermal growth factor receptor subject to retrograde trafficking drives epidermal growth factor-dependent migration

SUPPLEMENTARY MATERIALS

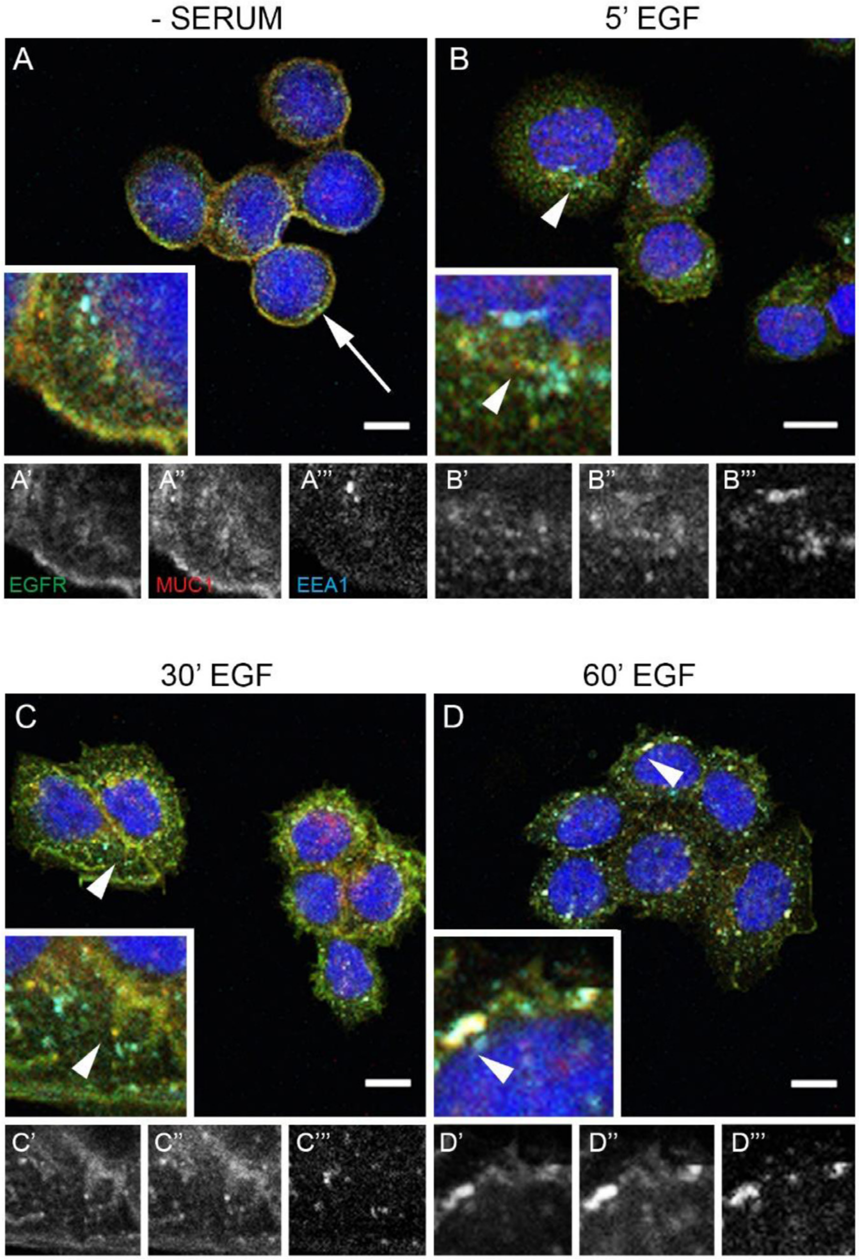

Supplementary Figure 1: EGFR and MUC1 colocalize together with EEA1 in MDA-MB-468 cells. (**A–D**) MDA-MB-468 cells were serum-starved overnight, treated with 20 ng/mL EGF (B–D), and incubated at indicated times at 37°C. Cells were incubated with either anti-EGFR 225 (green), anti-MUC1 Ab-5 (red), or anti-EEA1 H-300 (cyan), and mounted with DAPI (blue). Arrows indicate membrane localization and arrowheads indicate vesicular localization. Single prime (‘) images represent single channel EGFR of inset, double prime (“) images represent single channel MUC1 of inset, and triple prime (“‘) images represent single channel EEA1 of inset. Scale bar represents 10 μm.

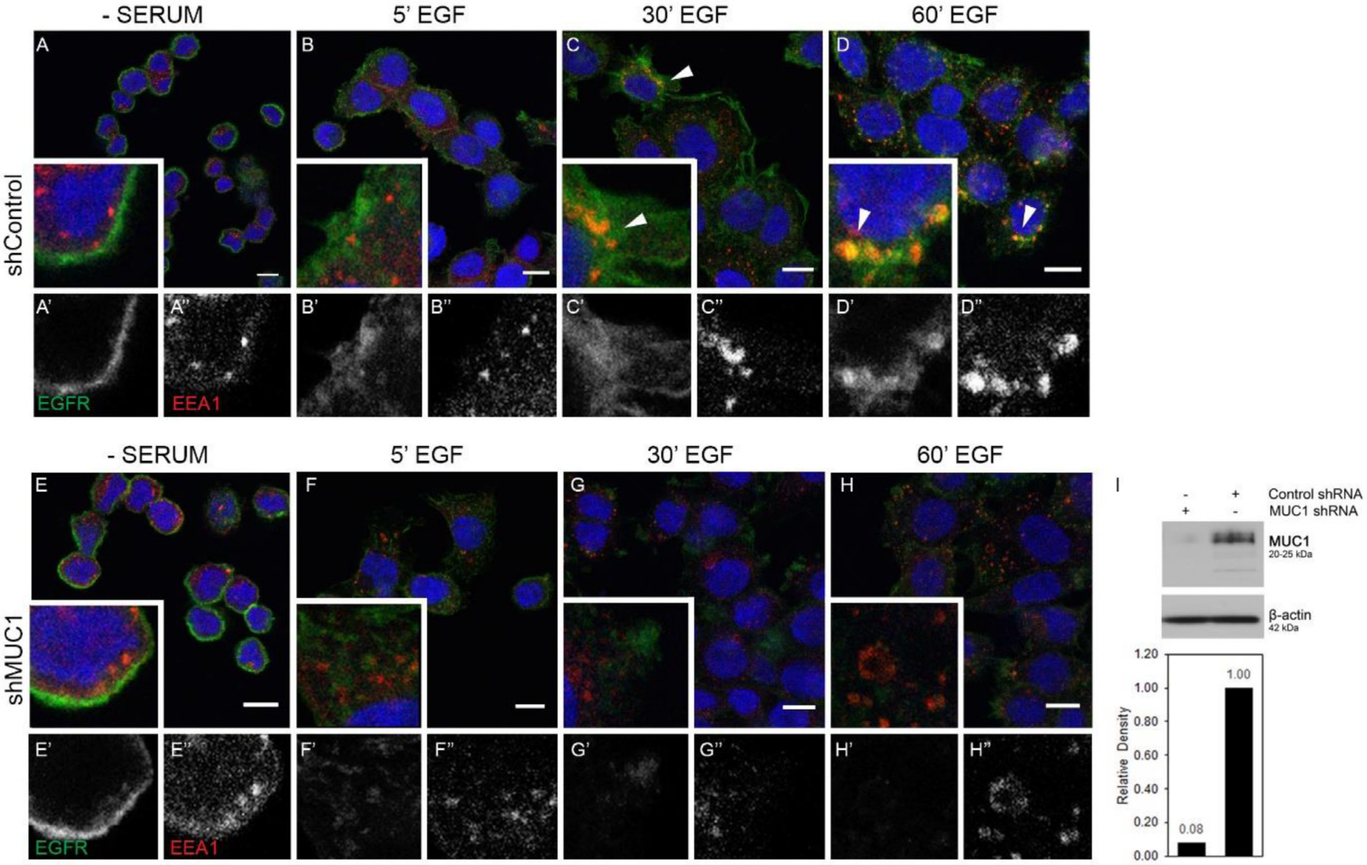

Supplementary Figure 2: EGFR association with EEA1 is prolonged and degradation is inhibited in the presence of MUC1 in MDA-MB-468 cells. (**A–H**) MDA-MB-468 cells were transfected with either control- or MUC1-specific shRNA, then stably selected with puromycin. Cells were serum-starved overnight, treated with 20 ng/mL EGF (B–D, F–H), and incubated at indicated times at 37°C. Cells were incubated with either anti-EGFR 225 (green) or anti-EEA1 H-300 (red) and mounted with DAPI (blue). Arrowheads indicate vesicular localization. Single prime (‘) images represent single channel EGFR of inset, double prime (“) images represent single channel EEA1 of inset. Scale bar represents 10 μm. (**I**) Protein lysates were collected from shRNA-treated BT20 cells and analyzed via immunoblot. Molecular weights are indicated on the right. Relative levels of MUC1 were quantified using ImageJ.

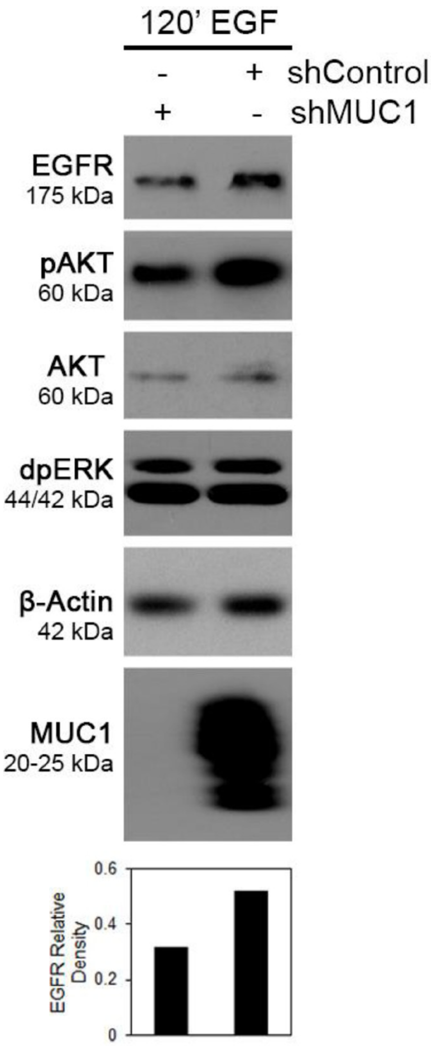

Supplementary Figure 3: MUC1 alters EGFR degradation and AKT pathway activity. Protein lysates were collected from BT20 cells +/– MUC1-directed shRNA after being subject to serum-starvation and treatment with 20 ng/mL of EGF. Lysates were analyzed via immunoblot using the indicated antibodies and molecular weights indicated on the left. Relative density was calculated using ImageJ.

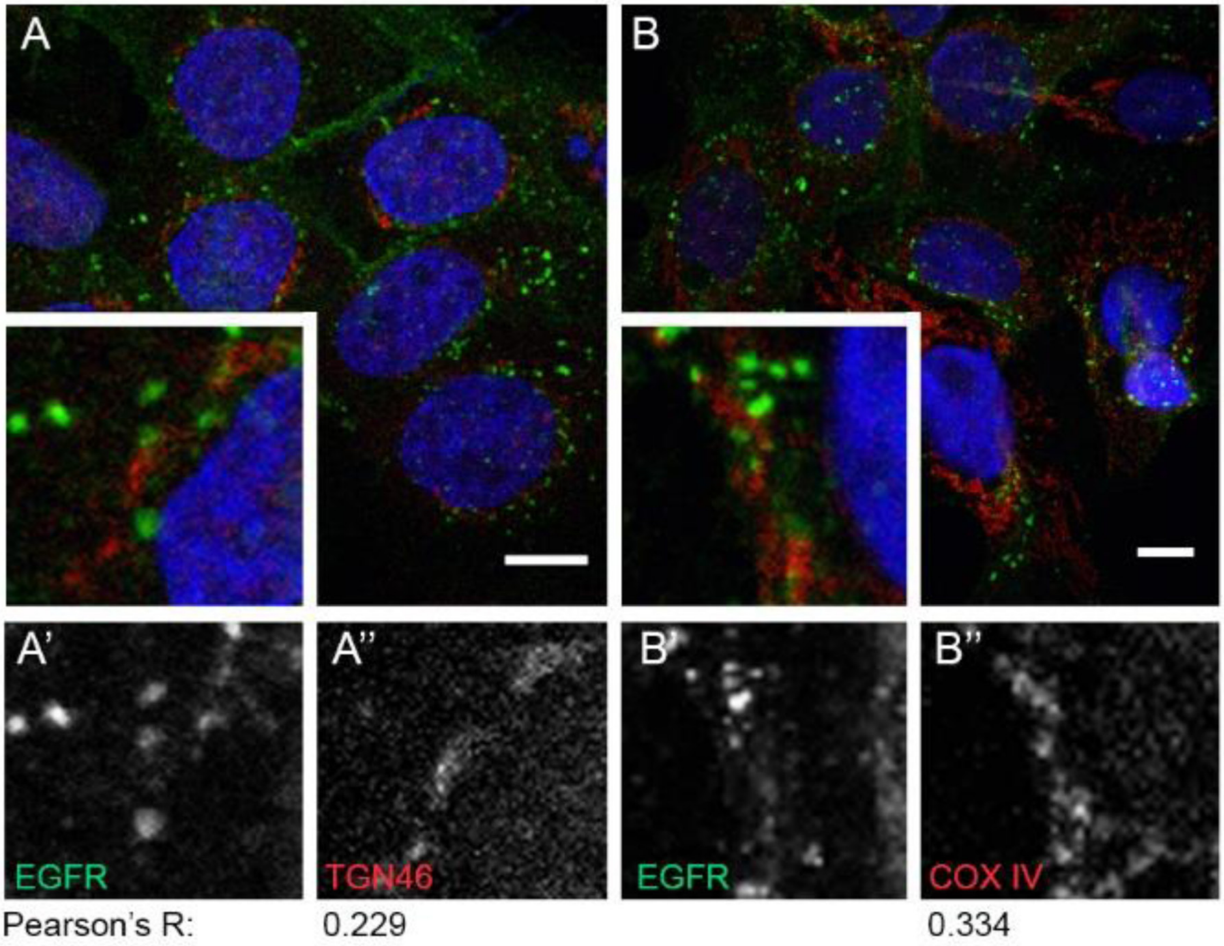

Supplementary Figure 4: EGFR does not colocalize with the trans-golgi marker TGN46 or the mitochondria in BT20 cells. (**A–B**) BT20 cells were treated with EGF and evaluated for localization of indicated proteins. Cells were incubated with either anti-EGFR 225 (green), anti-TGN46 (red) (A), or anti-COX IV (red) (B), and mounted with DAPI (blue). Single prime (') images represent single channel EGFR of inset, A'' represents single channel TGN46 of inset, B'' represents single channel COX IV of inset. Scale bar represents 10 µm. Quantification of Pearson's coefficient value r shown beneath images. *n* = 3.

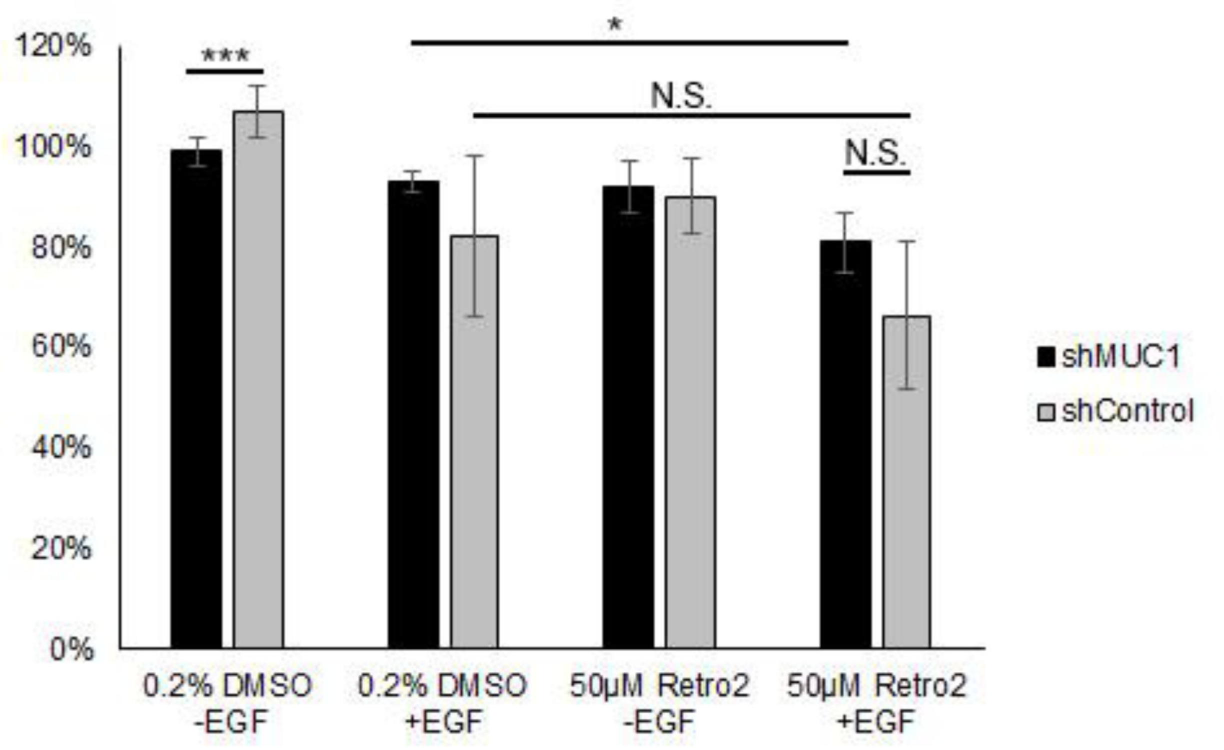

Supplementary Figure 5: Retro-2 does not alter cell viability in the presence or absence of MUC1. Cell viability assay performed in BT20 +/– MUC1 cells over 3 days comparing treatment with Retro-2 to DMSO in the presence or absence of 20 ng/mL EGF. Data shown represents mean +/– percent difference of assays performed in triplicate. $^{*}p < 0.05$, $^{***}p < 0.005$.

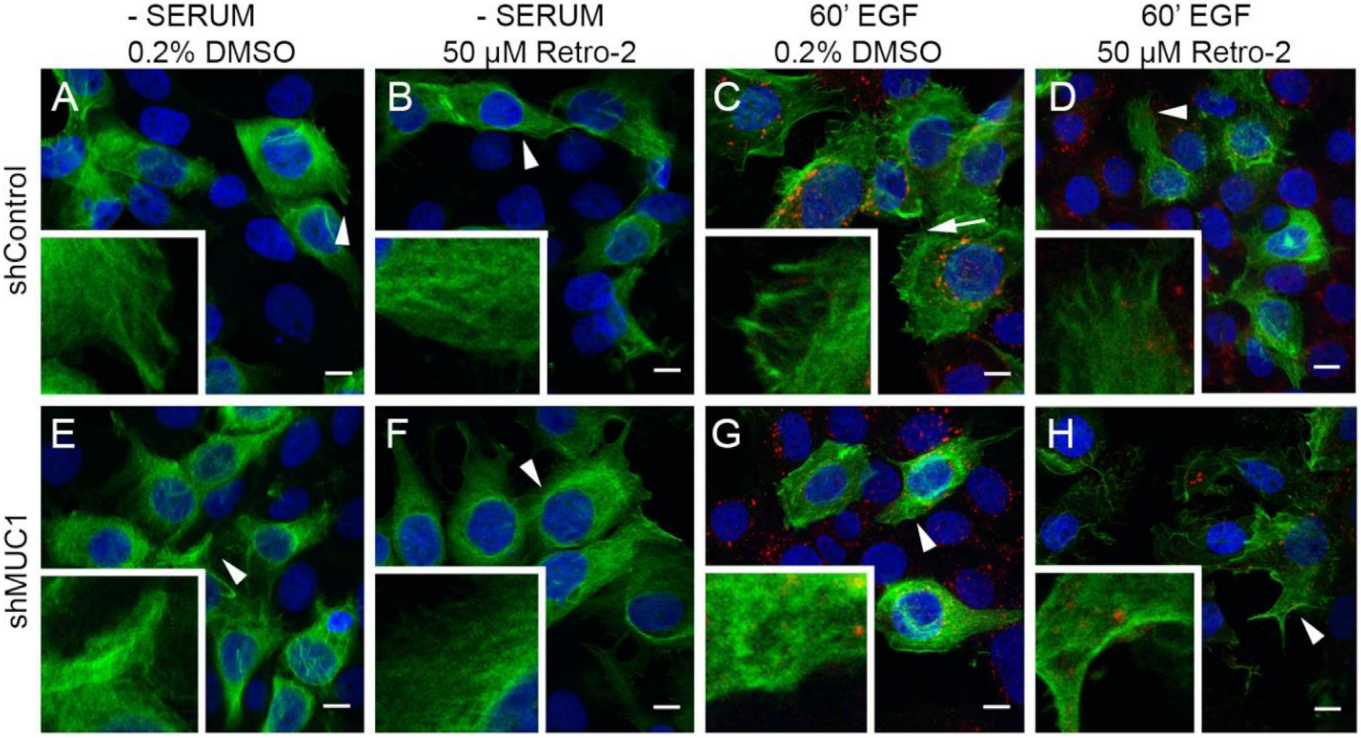

Supplementary Figure 6: Retro-2 inhibits for formation of actin protrusions. (**A–H**) BT20 +/– MUC1 cells were serum-starved, treated with DMSO or 50 μM Retro-2 followed by 200 ng/mL EGF-647 (red) (C, D, G, H), then incubated with DMSO or 50 μM Retro-2. Cells were incubated with phalloidin-488 (green) and DAPI (blue). Scale bar represents 10 μm. Arrows represent condensed actin filaments, arrowheads represent diffuse actin. *n* = 3 for all time points indicated.

Supplementary Video 1: EGFR traffics to the lysosome in the absence of MUC1. MDA-MB-468 cells were transfected with EGFR-GFP and transduced with MUC1-specific siRNA. Cells were incubated with Lysotracker Red, followed by 10 min treatment with EGF, prior to incubation with DMSO. Video captures 10–90 min post-EGF exposure. EGFR-GFP in green, Lysotracker Red in red, colocalization in yellow. See Supplementary_Video_1.
